# Expression, purification, and characterization of rhTyrRS

**DOI:** 10.1186/1472-6750-14-64

**Published:** 2014-07-15

**Authors:** Yongjiang Lang, Yanling Zhang, Ling Zhan, Zhe Feng, Xiushi Zhou, Min Yu, Wei Mo

**Affiliations:** 1 The Key Laboratory of Molecular Medicine, Ministry of Education, Fudan University, Shanghai, P.R. China; 2 The Department of Biochemistry and Molecular Biology, School of Basic Medical Sciences, Fudan University, Shanghai, P.R. China

**Keywords:** Recombinant human tyrosyl-tRNA synthetase, Expression of rhTyrRS, Protein purification, Biological activity assay

## Abstract

**Background:**

Aminoacyl-tRNA synthetases (AARSs) catalyze the first step of protein synthesis. Emerging evidence indicates that AARSs may have additional functions, playing a role in signal transduction pathways regulating thrombopoiesis and inflammation. Recombinant human tyrosyl-tRNA synthetase (rhTyrRS) is engineered with a single amino acid substitution that unmasks its cytokine activity. An industrial production method that provides high yield as well as high purity, quality, and potency of this protein is required for preclinical research.

**Results:**

We expressed codon-optimized rhTyrRS in *Escherichia coli* under fermentation conditions. Soluble protein was purified by a three-step purification method using cation exchange chromatography, gel filtration chromatography, and anion exchange chromatography. We also established a method to test the biological activity of rhTyrRS by measuring aminoacylation and IL-8 release in rhTyrRS-treated HL-60 cells.

**Conclusions:**

The characterization of purified rhTyrRS indicated that this protein can be used in pharmacodynamic and pharmacokinetic studies.

## Background

Aminoacyl-tRNA synthetases (AARSs) are enzymes that catalyze the essential first step of protein synthesis by covalently attaching each of the 20 standard amino acids to their cognate tRNA. Because the reactions require the capacity to recognize tRNAs as well as small chemicals such as amino acids and ATP, the structures of AARSs are well equipped for interacting with diverse molecules that may be associated with their functional versatility [1,2].

AARSs, such as glutamyl-prolyl-tRNA synthetase, glutaminyl-tRNA synthetase, and lysyl-tRNA synthetase, are now known to be multifunctional, participating in a variety of functions including transcription, translation, splicing, inflammation, thrombopoiesis, and apoptosis [3]. Human tyrosyl-tRNA synthetase (TyrRS) was the first example of a tRNA synthetase that played a role in cell signaling and thrombopoiesis. It has been demonstrated that human full-length TyrRS has no known cytokine activity [4]. However, proteolytic removal of the C-domain from TyrRS activates its cytokine function [1,2,5]. The N-terminal fragment, named mini-TyrRS, specifically stimulates migration of endothelial cells and polymorphonuclear leukocytes [1,2,6,7]. Mini-TyrRS has also been shown to activate thrombocytopoiesis in a chick chorioallantoic membrane thrombopoiesis assay [8].

As in some CXC chemokines, such as IL-8, an Glu-Leu-Arg motif embedded in the sequence of mini-TyrRS was shown by mutational analysis to be critical for its cytokine activity [9-11]. Although there is no crystal structure reported for full-length TyrRS, high-resolution crystal structures of mini-TyrRS and of the C-domain enabled reconstruction of the full-length enzyme, and has provided the model and structural basis for its cytokine activity [8,12]. It is predicted that the C-terminus of the full-length protein is tethered to the N-terminal region, thus preventing ELR substrate recognition [13,14].

Recombinant human TyrRS (rhTyrRS) is a full-length human tyrosyl-tRNA synthetase with a mutation at Y341A that blocks the H-bond between the ELR motif and Y341. As a result, the ELR motif is exposed, and mini-rhTyrRS-like activity can then promote thrombocytopoiesis [15,16].

As a potential thrombocytopoietic drug, large-scale expression and purification of rhTyrRS would be desirable. Preclinical testing involving *in vitro* and animal studies could then be carried out to evaluate its toxic and pharmacologic effects. In this study, rhTyrRS was expressed at a high level in *E. coli* and purified for future preclinical testing.

## Methods

### Cells and antibodies

The competent *E. coli* strain BL21 (F-ompT hsdS (rB-mB-) gal dcm; providded by aTyr Pharma) was used as the host for rhTyrRS expression. This strain was transformed with the pET24a inducible expression vector in which the His-tag sequence was deleted and the T7 promoter was replaced with a Tac promoter.

A mouse anti-human IL-8 monoclonal antibody (BC013615, Proteintech Group), rabbit anti-human IL-8 polyclonal antibody (BC013615, Proteintech Group), and goat anti-rabbit IgG-HRP SC-2004 antibody (D2111, Santa Cruz Biotechnology) were used.

### Growth conditions

A clone with a high rhTyrRS expression level was cultivated in LB agar and M9CA medium (10 g glucose, 6 g Na_2_HPO_4_, 35 g KH_2_PO_4_, 2.93 g NaCl, 0.4 g NH_4_Cl, 1.2 g MgSO_4_, and 1 mL trace element solution per liter). Trace element solution (1 L) consisted of 2.8 g FeSO_4_ · 7H_2_O, 2 g MnCl_2_ · 4H_2_O, 2.8 g CoSO_4_ · 7H_2_O, 1.5 g CaCl_2_ · 2H_2_O, 0.2 g CuCl_2_ · 2H_2_O, and 0.3 g ZnSO_4_ · 7H_2_O. Feeding solutions were 50% glycerol, 250 g/L glucose, and 100 g/L yeast extract. The glucose and MgSO_4_ solutions were sterilized separately. Kanamycin sulfate was added to a final concentration of 100 μg/mL in both the M9CA and feeding solutions.

Fermentation was performed in a 5-L Bioflo 3000 fermenter (New Brunswick Scientific, New Brunswick, NJ, USA) with automated control of: the pH at 7.0 by the addition of ammonium hydroxide, dissolved O_2_ at 70% by providing pure oxygen, and agitation at 700 rpm. Air was provided at a flow rate of 4.0 L/min and the temperature was controlled at 30°C. Fermentation was conducted according to the process developed by Shiloach *et al.* (1996) [17]. The batch phase ends when cells have used up the available glucose. The best indications that the batch phase has ended include a sharp decrease in stirrer speed and an increase in pO_2_. The bacterial concentration was measured off-line by the optical density at 600 nm and induced with 0.5 mM isopropylthio-β-galactoside (IPTG) once it reached an OD600 of 30 (~10 h). After 6 h of 0.5 mM IPTG induction, the cells were harvested by centrifugation at 6,000 × *g* for 30 min. The cell pellet was stored at -70°C.

### Cell lysis

Harvested cell pellets were resuspended in 10 volumes of 20 mmol/L HAc-NaAc buffer (pH 6.0) and subjected to two cycles of microfluidization at 1000 bar. The crude extract was then centrifuged at 10,000 × *g* for 60 min.

### Cation exchange chromatography

The clarified supernatant was loaded onto a SP Sepharose Fast Flow column (GE) that was pre-equilibrated with 20 mmol/L HAc-NaAc buffer (pH 6.0). The bound proteins were eluted with a linear NaCl gradient (0 to 1 mol/L). Fractions containing rhTyrRS were pooled and analyzed by SDS-PAGE.

### Gel filtration

The pooled fractions were loaded onto a Sephadex-G50 column (GE) pre-equilibrated with 20 mmol/L phosphate buffer (PB; pH 7.0) and rhTyrRS was washed with 20 mmol/L PB at 5 mL/min. Fractions were collected from the column and analyzed by SDS-PAGE.

### Anion exchange chromatography

The diluted product solution was loaded onto a 50-mL Q Sepharose Fast Flow column (GE) pre-equilibrated with 20 mmol/L PB (pH 7.0). RhTyrRS was eluted with a linear NaCl gradient (0 to 1 mol/L) and identified by SDS-PAGE via Coomassie blue staining.

### SDS-PAGE and sequencing of the N-terminal amino acids

Electrophoresis was carried out in 1-mm-thick gels using BioRad MiniGel apparatus. Coomassie staining was performed as previously described, except that microwave heating was used at each staining step to reduce the total staining and destaining procedure time to 30 min. The protein concentration was measured using BSA as a standard.

The sequence of the N-terminal amino acids of purified rhTyrRS was determined using a protein sequencer (PPSQ-33A, USA).

### Western blotting

Proteins resolved in a pre-cast Bis-Tris gel (BioRad) were electrotransferred to a PVDF membrane followed by blocking in 10% BSA solution prepared in TBST (Tris-buffered saline with 0.1% Tween-20). The membrane was then incubated with anti-rhTyrRS monoclonal antibody (1/5000) for 90 min at room temperature. After washing, the membrane was incubated with peroxidase-conjugated goat anti-mouse IgG (1/100,000) for 60 min at room temperature. All antibody incubations and washing steps were carried out in TBST. The immunoreactive bands were visualized with a Western Blot kit (Thermo 34077).

### Reversed-phase HPLC

Reversed-phase HPLC was used to study the purity of rhTyrRS. A Higgins Proto 300 C_4_ column (Waters 150 × 4.6 mm) was used at a flow rate of 1 mL/min and at a temperature of 40°C. The column was eluted with a linear 30-min gradient from 5% B to 95% B (A = 0.1% Trifluoroacetic Acid (TFA) in HPLC water; B = 0.1% TFA in acetonitrile) and monitored by absorbance at 215 nm with a total run time of 40 min.

### Mass spectrometry analysis

The purified protein was analyzed by mass spectrometry. Molecular weight measurements were made by LC-MS with a quadrupole-time-of-flight mass spectrometer at the Fudan University Institute of Biomedical Science.

### Aminoacylation assay

Aminoacylation activity was determined at ambient temperature in 150 mmol/L Tris–HCl (pH 7.5), 150 mmol/L KCl, 10 mmol/L MgCl_2_, 10 mmol/L β-mercaptoethanol, 4 mmol/L ATP, and 10 mmol/L tyrosine (including 3 mmol/L [^3^H]-tyrosine, GE Healthcare). Human tRNATyr was prepared as previously described [12]. Before each assay, 100 mmol/L human tRNATyr was annealed by heating at 65°C for 5 min and cooled to room temperature. Reactions were initiated by the addition of rhTyrRS (10 nmol/L) to the reaction mixture. Aliquots were taken at fixed intervals and spotted onto Whatman filter discs saturated with 5% trichloroacetic acid and dried. The filters were placed into ice-cold 5% trichloroacetic acid and washed three times with fresh 5% trichloroacetic acid at 4°C and once with 95% ethanol. The level of tRNA aminoacylation was then quantitated by liquid scintillation counting.

### Biological activity assay

RhTyrRS activity was tested using ELISA to measure IL-8 secretion by rhTyrRS-treated HL-60 cells. Cells were thawed from vials and cultured according to the American Type Culture Collection instructions, except that RPMI-1640 (ATCC 30–2001) was supplemented with 10% heat-inactivated fetal bovine serum (Life Technologies, formerly Invitrogen 10082–147) and used as growth medium. Cells were plated in a 96-well plate at a density of 1 × 10^6^ cells per 1 mL of medium on the day of the assay. The rhTyrRS was added immediately after cell plating. Cells were plated at 10^8^ μl^-1^ per well. The protein was diluted in sterile phosphate-buffered saline (PBS; Life Technologies, formerly Invitrogen 10010–023) to a concentration of 10 μmol/L (10× protein stock). The 10× protein stock was serially diluted and 12 μL was added to each well such that the final protein concentrations were 500 nmol/L, 250 nmol/L, 125 nmol/L, 62.5 nmol/L, 31.25 nmol/L, and 15.625 nmol/L. PBS was used as a control. Cells were incubated at 37°C and 5% CO_2_ for 24 h. The 96-well plate was then spun for 10 min at room temperature in a swinging bucket centrifuge at 1,000 × *g*. The plate was tilted at a 45° angle and 100 μL of media per well was transferred to a new 96-well plate. The amount of secreted IL-8 protein in the media was then determined by sandwich ELISA.

## Results

### rhTyrRS expression and purification

**Figure 1:**
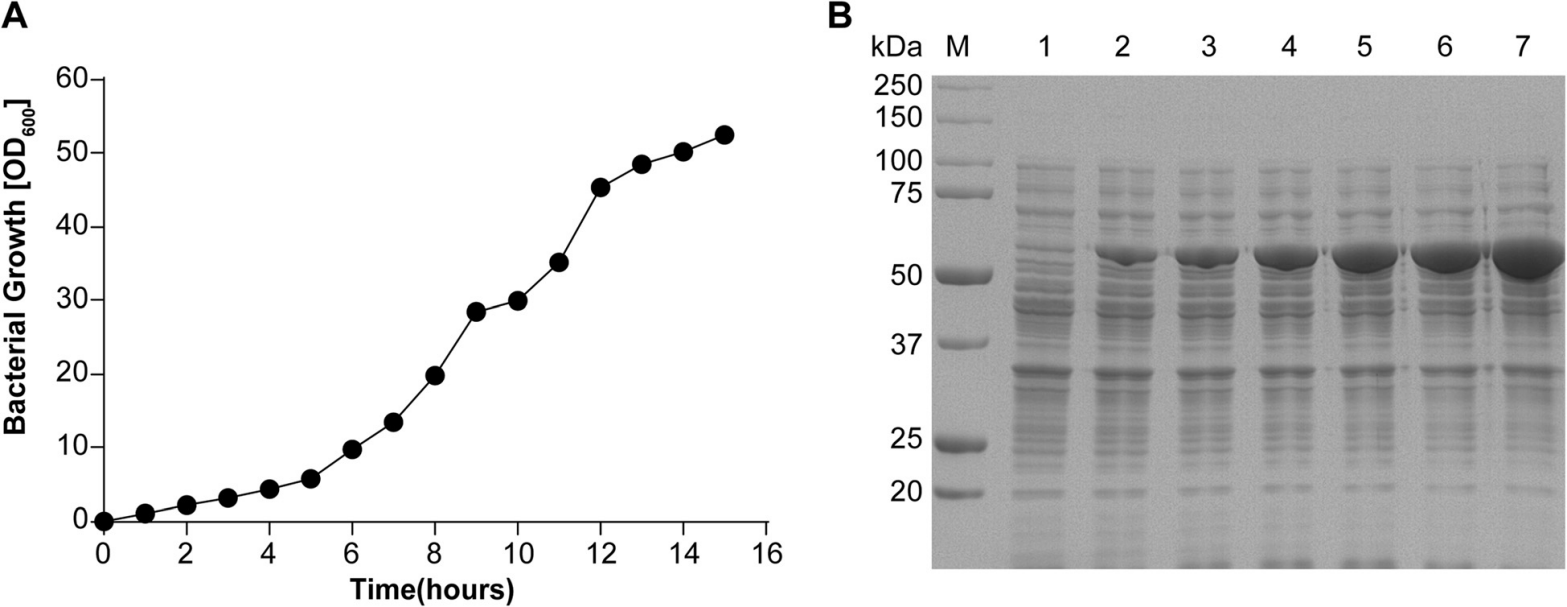
**Expression and purification of rhTyrRS. A**: Fed-batch growth curve of BL21 cells expressing rhTyrRS. A 5-L fermentor was inoculated with a 150-mL seed culture at time 0 and samples were drawn at regular intervals to monitor the optical density at 600 nm. rhTyrRS expression was induced with IPTG at approximately OD600 = 30. **B**: SDS-PAGE analysis of rhTyrRS expressed by fed-batch fermentation. Lane 1, standard protein marker (20–250 kD); Lane 2, uninduced BL21 cells; Lanes 3–8, BL21 cells induced with 0.5 mM IPTG for 1, 2, 3, 4, 5, and 6 h.

**Figure 2:**
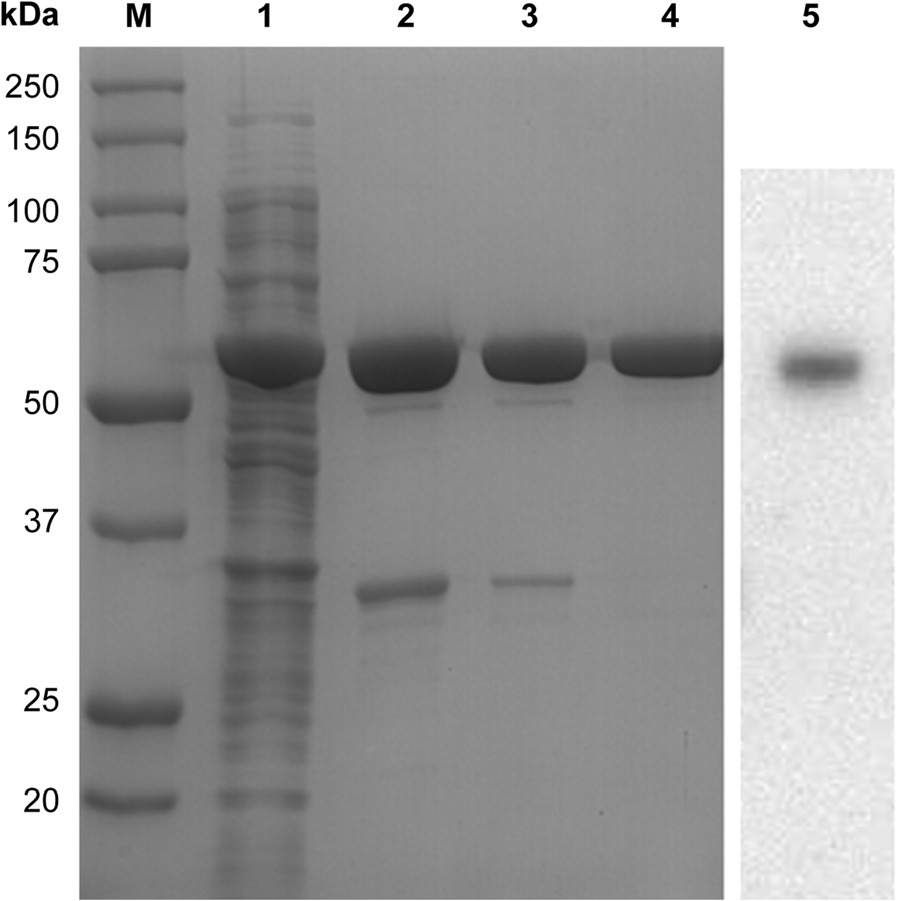
**Purification of rhTyrRS using a three-step procedure.** M, standard protein marker (20–250 kD). Lane 1, soluble lysate from induced cells; Lane 2, cation exchange chromatography wash fraction; Lane 3, gel filtration wash fraction; Lane 4, anion exchange chromatography wash fraction; Lane 5, western blot assay of rhTyrRS.

To evaluate the long-term stability of the plasmid/strain combination, bacteria were first grown in shake flasks for 25 generations by repeated dilution before the analysis of rhTyrRS expression. A typical growth curve is shown in Figure 1A. SDS-PAGE analysis of expressed rhTyrRS is shown in Figure 1B. The expression level after 25 generations was comparable to that produced by the cells after five generations. Approximately 1.5–2.0 g/L of rhTyrRS was produced under these growth conditions. When rhTyrRS was expressed in *E. coli* strain BL21 using M9CA medium and fed-batch methodology, approximately 50% of the protein was soluble.After 6 h of IPTG induction, cells were harvested by centrifugation and lysed by two cycles of microfluidization. The concentrated media were subjected to cation exchange, gel filtration, and anion exchange chromatography, yielding rhTyrRS of up to 97% purity. RhTyrRS was eluted with 0.25 mol/L NaCl-PB via cation exchange, then subjected to gel filtration to exchange the buffer to PB; all pooled fractions were then loaded onto a 50 mL Q Sepharose Fast Flow column pre-equilibrated with 20 mmol/L PB (pH 7.0). RhTyrRS was eluted with less than 0.1 mol/L NaCl-PB during anion exchange. Around 1.0 g of purified rhTyrRS was generated from 3 L of culture. The products from the optimized rhTyrRS purification process, consisting of cell lysis followed by cation exchange chromatography, gel filtration, and anion exchange chromatography, are shown in Figure 2.

### rhTyrRS characterization

The identity of rhTyrRS was verified by Edman degradation to compare the N-terminal sequence of rhTyrRS to the expected mini-TyrRS sequence. Up to 15 amino acids from the N-terminus of rhTyrRS were consistently identical to the reference sequence: Met-Gly-Asp-Ala-Pro-Ser-Pro-Glu-Glu-Lys-Leu-His-Leu-Ile-Trp. We also sought to confirm the identity of rhTyrRS by resolving the sample (s) in parallel with a reference standard by SDS-PAGE followed by western blotting using a polyclonal antibody (Figure 2).

### Reversed-phase HPLC

**Figure 3:**
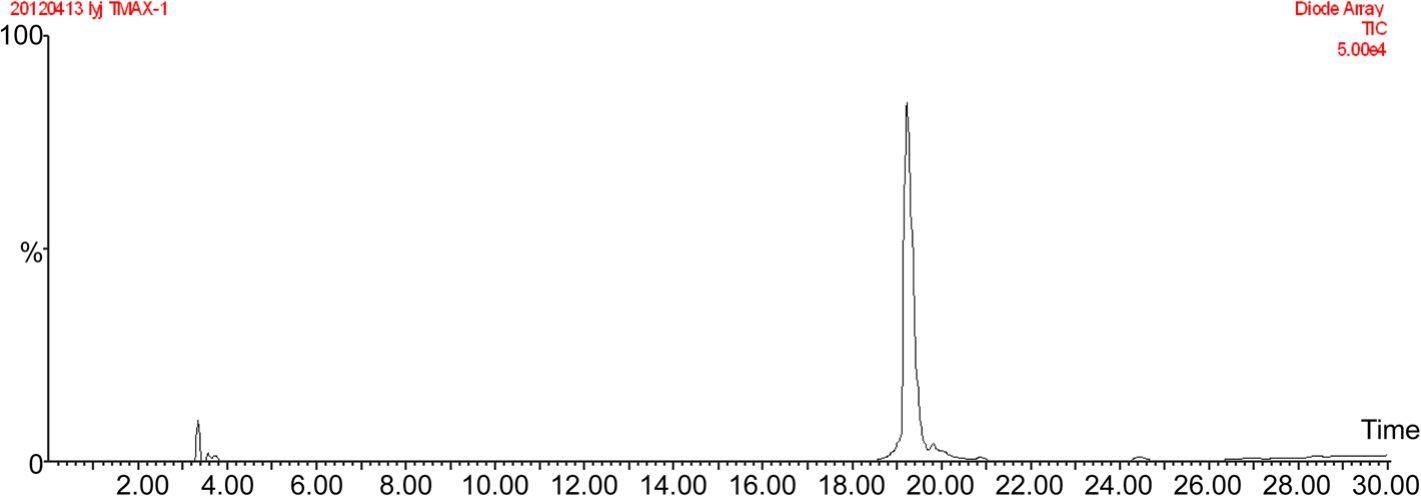
**Purity analysis of rhTyrRS by HPLC.** A single symmetrical peak with a 97% purity was indicated in the HPLC analytical chart.

A reversed-phase HPLC was developed as a purity indication assay. Figure 3 shows the analysis of rhTyrRS purified using chromatography steps. This method will be transferred to the contract manufacturer organization or its approved vendor, as appropriate. The retention time of rhTyrRS in the C4 column was 19.5 min with purity up to 97%.

### Mass spectrometry analysis

**Figure 4:**
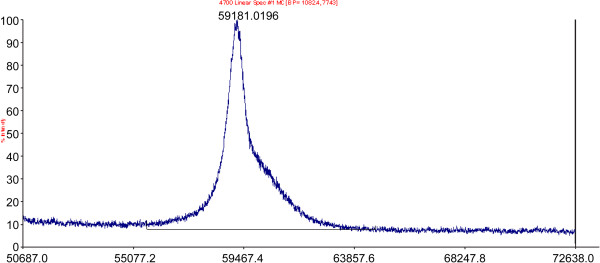
**Molecular mass determination of purified rhTyrRS by LC-MS.** Peaks corresponding to various protonated rhTyrRS species are marked. The mass analysis determined that rhTyrRS has a molecular weight of 59.18 kD.

An accurate mass can be detected by intact mass analysis using LC-MS. Peaks corresponding to various protonated rhTyrRS species are marked. Mass analysis determined that rhTyrRS has a molecular weight of 59.18 kD (Figure 4). This is identical to that identified by SDS-PAGE.

### Aminoacylation assay

**Figure 5:**
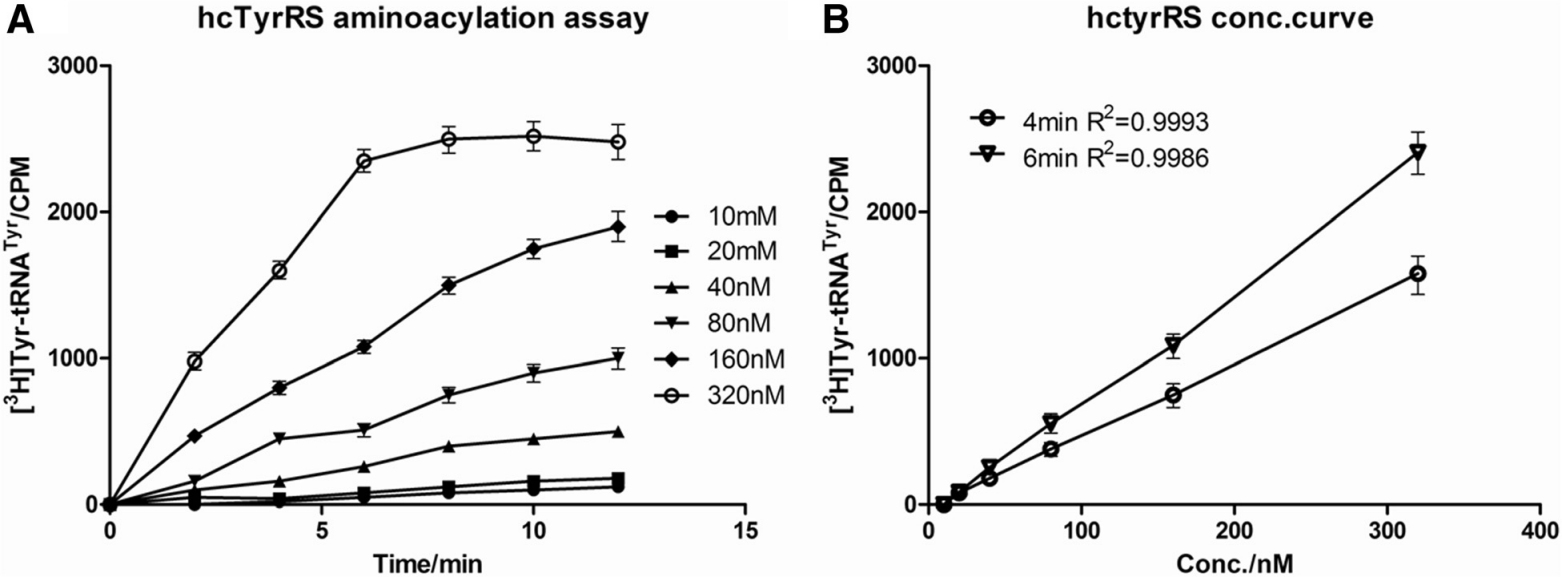
**rhTyrRS is a functional aminoacyl-tRNA synthetase. A**: The ability of rhTyrRS to charge tRNA with ^3^H-labeled tyrosine was tested *in vitro* as a function of rhTyrRS concentration and time. The following concentrations of rhTyrRS were tested: 320 nM, 160 nM, 80 nM, 40 nM, 20 nM, and 10 nM. **B**: The ability of rhTyrRS to charge tRNA after 4 and 6 min.

To test whether rhTyrRS retained the ability to aminoacylate its cognate tRNA molecule, we took advantage of the inherent synthetase activity of rhTyrRS to verify proper protein folding and assess biological activity during the expression and purification process. The incorporation of ^3^H tyrosine into tRNA is a well-established and reproducible assay [4,5] (Figure 5).

### Biological activity assay

**Figure 6:**
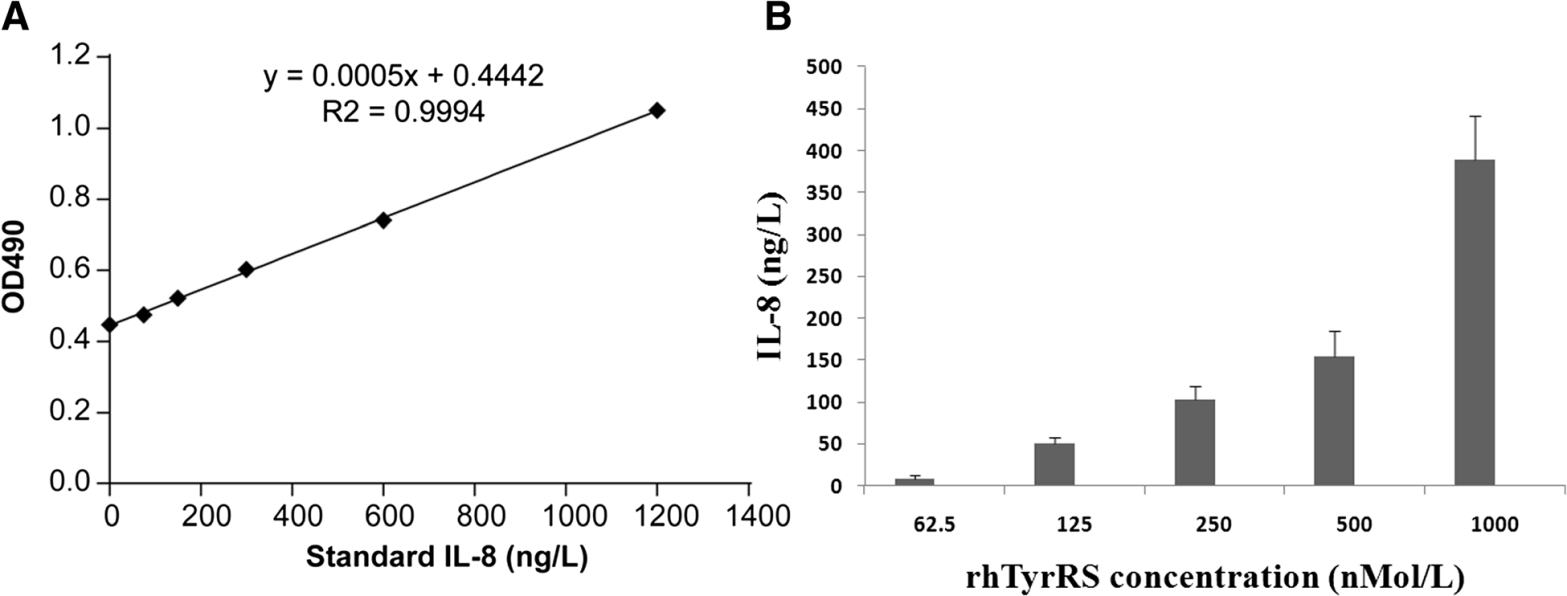
Dose-dependent induction of interleukin-8 (IL-8) production in HL-60 cells by rhTyrRS.

We used a sandwich ELISA to test IL-8 released by HL-60 cells in the presence of rhTyrRS. The standard IL-8 concentrations used for ELISA were 1200 ng/mL, 600 ng/mL, 300 ng/mL, 150 ng/mL, 75 ng/mL, and 32.5 ng/mL as shown in Figure 6A, and R^2^ was 0.9924. Figure 6B shows the amounts of IL-8 released by HL-60 cells incubated in the presence of rhTyrRS at concentrations between 62.5 nmol/L and 1000 nmol/L, based on the standard IL-8 shown in Figure 6A.

## Discussion and conclusions

To facilitate the use of rhTyrRS proteins in regulating thrombopoietic activity and to enable structural characterization of this protein, we established a system for the production of large amounts of rhTyrRS. A purity of approximately 97% was routinely achieved with <2% individual contaminants and little to no aggregation.

The final, optimized process consists of cell lysis followed by a sequence of three chromatography steps. The first step (cation exchange) captures rhTyrRS while contaminants flow through, the second step (gel filtration) exchanges the buffer to PB, and the last step (anion exchange) removes the remaining process-related impurities. The final concentration is 1 mg/ml in PBS (pH 7.4). Preliminary characterization of rhTyrRS was performed using western blotting, HPLC, mass spectrometry, and N-terminal sequencing to verify the identity of the protein.

The ability of the purified rhTyrRS to aminoacylate its cognate tRNA molecule was also tested, and analysis of the inherent synthetase activity of rhTyrRS was performed to verify proper protein folding and assess biological activity during the expression and purification processes. Kinetic parameters from different preparations of rhTyrRS will be compared with a well-characterized reference standard. Purification lots that are within the established specifications will be deemed acceptable for release. This assay is still under development but a representative example of the linearity of product accumulation over time is shown below with a range of rhTyrRS concentrations.

The Y341A substitution activated full-length TyrRS for stimulation of cell proliferation. Similar results were observed in an endothelial cell migration assay. In these experiments, wounds were created in monolayers of human umbilical vein endothelial cells [9]. We predict that rhTyrRS not only has IL-8-like cytokine activity, but also can enhance IL-8 production from monocytes [16]. The biological assays of HL-60 stimulation with rhTyrRS presented here demonstrate that this may be the case. The findings presented here will be important for future preclinical studies.

## Competing interests

The authors declare that they have no competing interests.

## Authors’ contributions

YL, LZ and XZ performed the protein expression and purification; YZ and ZF analyzed the protein activity; YL and WM designed the experiments, analyzed data, and wrote the paper; MY supervised the experiments and designed the study. All authors read and approved the final manuscript.

## Authors’ information

Yongjiang Lang and Yanling Zhang are first author.
